# Dynamic Changes in High-Sensitivity Cardiac Troponin I in Response to Anthracycline-Based Chemotherapy

**DOI:** 10.1016/j.clon.2019.11.008

**Published:** 2020-05

**Authors:** E. Tzolos, P.D. Adamson, P.S. Hall, I.R. Macpherson, O. Oikonomidou, M. MacLean, S.C. Lewis, H. McVicars, D.E. Newby, N.L. Mills, N.N. Lang, P.A. Henriksen

**Affiliations:** † BHF Centre for Cardiovascular Sciences, University of Edinburgh, Edinburgh, UK; ‡ Christchurch Heart Institute, University of Otago, Christchurch, New Zealand; § Cancer Research UK, Edinburgh Centre, MRC Institute Genetics and Molecular Medicine, University of Edinburgh, Edinburgh, UK; ¶ Institute of Cancer Sciences, University of Glasgow, Glasgow, UK; || Edinburgh Clinical Trials Unit, University of Edinburgh, Edinburgh, UK; ∗∗ Usher Institute of Population Health Sciences and Informatics, University of Edinburgh, Edinburgh, UK; †† Department of Cardiology, Queen Elizabeth University Hospital, Glasgow, UK

**Keywords:** Anthracycline, breast cancer, cardiotoxicity, high-sensitivity cardiac troponin, left ventricular ejection fraction

## Abstract

**Aims:**

Treatment advances have improved cancer-related outcomes and shifted interest towards minimising long-term iatrogenic complications, particularly chemotherapy-related cardiotoxicity. High-sensitivity cardiac troponin I (hs-cTnI) assays accurately quantify very low concentrations of plasma troponin and enable early detection of cardiomyocyte injury prior to the development of myocardial dysfunction. The profile of hs-cTnI in response to anthracycline-based treatment has not previously been described.

**Materials and methods:**

This was a multicentre prospective observational cohort study. Female patients with newly diagnosed invasive breast cancer scheduled to receive anthracycline-based (epirubicin) chemotherapy were recruited. Blood sampling was carried out before and 24 h after each cycle. Hs-cTnI concentrations were measured using the Abbott ARCHITECT_*STAT*_ assay.

**Results:**

We recruited 78 women with a median (interquartile range) age of 52 (49–61) years. The median baseline troponin concentration was 1 (1–4) ng/l and the median cumulative epirubicin dose was 394 (300–405) mg/m^2^. Following an initial 33% fall 24 h after anthracycline dosing (*P* < 0.001), hs-cTnI concentrations increased by a median of 50% (*P* < 0.001) with each successive treatment cycle. In total, 45 patients had troponin measured immediately before the sixth treatment cycle, 21 (46.6%) of whom had hs-cTnI concentrations ≥16 ng/l, indicating myocardial injury. Plasma hs-cTnI concentrations before the second treatment cycle were a strong predictor of subsequent myocardial injury.

**Conclusions:**

Cardiotoxicity arising from anthracycline therapy is detectable in the earliest stages of breast cancer treatment and is cumulative with each treatment cycle. This injury is most reliably determined from blood sampling carried out before rather than after each treatment cycle.

## Highlights

• Plasma hs-cTnI concentrations showed an anthracycline dose-dependent increase.
• Hs-cTnI could act as an early and sensitive anthracycline cardiotoxicity prediction tool.
• Hs-cTnI measurement should happen before rather than after each cycle.

## Introduction

Anthracyclines are associated with cardiotoxicity and the development of heart failure [1]. The incidence of congestive heart failure during doxorubicin treatment in three studies comprising 630 breast and lung cancer patients rose exponentially from 5% with a cumulative dose of 400 mg/m^2^ to 48% with 700 mg/m^2^ [2]. With contemporary lower dose regimens, only a minority of patients develop clinical evidence of heart failure. For example, a study of cardiotoxicity in 2625 patients (74% women; 51% breast cancer and 28% non-Hodgkin lymphoma) reported lower rates of symptomatic heart failure [3]. Cardiotoxicity was defined as >10 percentage point decline in left ventricular ejection fraction (LVEF) and a drop to <50%. The incidence of cardiotoxicity was 9%, with 98% of cases developing in the first year. In 82% of cases the cardiotoxicity was either asymptomatic or only mildly symptomatic [3]. The recent PRADA Study (Prevention of cardiac dysfunction during an Adjuvant Breast Cancer Therapy) investigated breast cancer patients receiving anthracycline and trastuzumab. In PRADA, no patients developed symptomatic heart failure and the mean drop in cardiac ejection fraction was only 2.6 percentage points [4].

The development of asymptomatic myocardial dysfunction has important implications with regards to cancer treatment options [3,4]. Currently, the detection of cardiac toxicity focusses on cardiac imaging, including quantification of the LVEF [5]. This approach is operator dependent, expensive and inefficient, as most patients are not affected and receive unnecessary scans. Cardiac imaging is also relatively insensitive to early toxicity and identifies only advanced myocardial injury once changes in systolic or diastolic function have occurred. Early recognition of cardiac toxicity may allow targeted intervention in high-risk individuals before clinically significant deterioration in cardiac function. Although there are monitoring guidelines for cardiotoxicity, the optimal timing for the early detection of subclinical cardiotoxicity is still ambiguous [5,6].

Cardiac troponin I and T are specific markers of cardiomyocyte injury. Previous studies using contemporary sensitivity assays have shown a relationship between troponin rise during and after anthracycline chemotherapy and the subsequent development of left ventricular dysfunction [[7], [8], [9]]. Patients with no troponin concentration increase during or after chemotherapy are in a low-risk group for the development of cardiotoxicity [7,10].

The development of high-sensitivity cardiac troponin (hs-cTn) assays has allowed the accurate quantification of very low concentrations and the detection of circulating levels in over 50% of the healthy population [11,12]. Our group has shown the powerful predictive value of small changes in plasma hs-cTnI in a range of conditions affecting the heart, including coronary heart disease [[12], [13], [14]], aortic valve disease [15] and chronic obstructive airways disease [16]. To date, few studies have examined hs-cTn measurement in patients receiving anthracycline chemotherapy. In the PRADA study, hs-cTnI and hs-cTnT concentrations exhibited an anthracycline dose-dependent increase from baseline, after the completion of chemotherapy [4,17]. Given the low-level troponin concentration increases that occur after anthracycline administration, hs-cTn testing may better inform risk stratification for the development of left ventricular dysfunction. Furthermore, improved understanding of the kinetics of hs-cTn release and optimal timing for blood sampling during anthracycline chemotherapy will allow the implementation of surveillance protocols for cardiotoxicity.

The aim of this current study was to characterise the change in plasma hs-cTnI concentration in patients with breast cancer receiving anthracycline chemotherapy. This work was carried out to inform the protocol of the ongoing Cardiac CARE study (ISCRTN24439460), a randomised multicentre study using hs-cTnI to identify cancer patients at high risk of developing anthracycline cardiotoxicity.

## Materials and Methods

### Study Design and Participants

Between January 2016 and August 2017, consecutive patients with newly diagnosed breast cancer scheduled for anthracycline treatment in the Edinburgh Cancer Centre and the Beatson West of Scotland Cancer Centre (Glasgow) were approached to take part. All patients had a baseline LVEF >50% on echocardiography or radionucleotide ventriculography. Patients were planned to receive adjuvant chemotherapy with the FEC 80 regimen (six cycles of 5-fluorouracil, epirubicin 80 mg/m^2^ and cyclophosphamide) or adjuvant/neoadjuvant chemotherapy FEC 100 (three cycles of 5-fluorouracil, epirubicin 100 mg/m^2^ and cyclophosphamide) administered intravenously over 21-day cycles. FEC 100 was typically followed by docetaxel or paclitaxel with or without trastuzumab depending on HER2 status. However, troponin results are only reported for the anthracycline-containing cycles of treatment.

We measured hs-cTnI concentration at baseline (24 h before cycle 1), during (24 h after anthracycline infusion) and before each treatment cycle.

A high-sensitivity assay (ARCHITECT_*STAT,*_ Abbott Laboratories) was used to measure cardiac troponin I concentrations. This assay has a limit of detection of 1.2 ng/l and an upper reference limit (99th centile) of 34 ng/l in men and 16 ng/l in women. It has a coefficient of variation of 23% at the limit of detection (1.2 ng/l) and <10% at 4.7 ng/l [11,12].

All statistical analyses, including correlation and non-linear regression, were carried out with GraphPad Prism version 8.0.1 (GraphPad Software, San Diego, California, USA). *P*-value was calculated using one-way ANOVA.

All participants provided informed consent before participation in the study.

## Results

We enrolled 78 women (median age 52 years, interquartile range [IQR] 49–61 years) with low rates of cardiovascular risk factors (Table 1).

**Table 1:** Baseline characteristics

	Total cohort
*n*	78
Age (years)	52 [49–61]
Body mass index (kg/m^2^)	28.2 ± 5.7
Hypertension	17 (16)
Smoking habit	
Current smoker	13 (13)
Ex-smoker	31 (30)
Never smoked	60 (58)
Diabetes mellitus	6 (6)
Baseline LVEF (%)	63.9 ± 6.8
Baseline hs-cTnI (ng/l)	1.0 [1.0, 4.0]
Cumulative epirubicin dose (mg/m^2^)	394.1 [299.7, 405.4]

Hs-cTnI, high-sensitivity cardiac troponin I; LVEF, left ventricular ejection fraction.

Data are mean ± standard deviation, median [interquartile range] or value (%).

**Fig 1:**
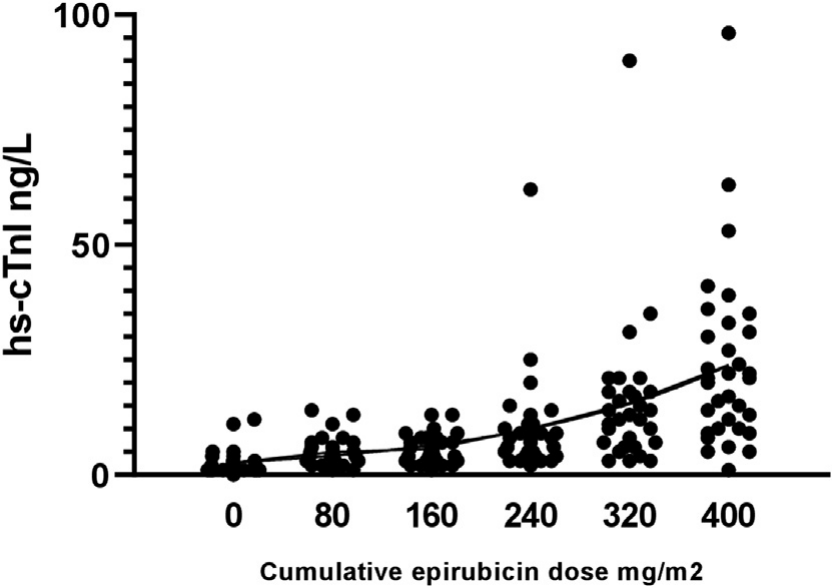
Correlation between circulating high-sensitivity cardiac troponin I (hs-cTnI) concentration and cumulative epirubicin dose. Scatterplot of individual patient hs-cTnI concentrations according to cumulative epirubicin dose (*n* = 78).

The median troponin concentration before cycle 1 was 1 ng/l (IQR 1–4 ng/l). Pre-treatment hs-cTnI concentrations increased with each cycle and were strongly correlated with the cumulative epirubicin dose (*r*^2^ = 0.9036; *P* = 0.036) (Figure 1).

**Fig 2:**
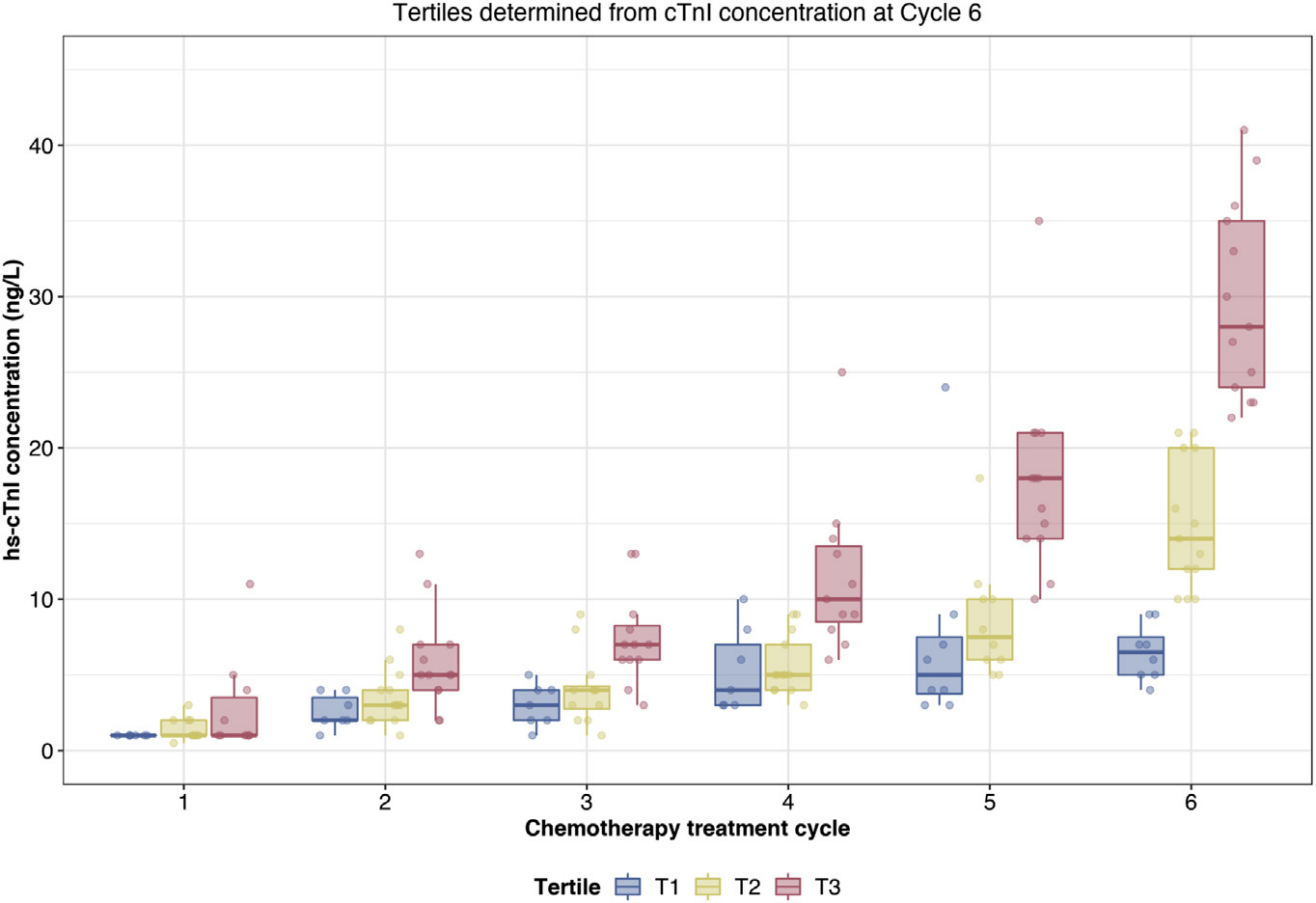
High-sensitivity cardiac troponin I (Hs-cTnI) concentration tertile plot for patients receiving six cycles of epirubicin. Plot of hs-cTnI concentration (median and interquartile range) tertiles according to concentration before sixth treatment cycle. The same patients in each tertile from this final cycle are plotted in preceding cycles (*n* = 45).

Forty-five patients receiving the FEC 80 regimen had hs-cTnI quantification before the sixth cycle and this group was divided into tertiles according to hs-cTnI concentration before the sixth epirubicin cycle (Figure 2). Baseline characteristics, including troponin concentration 1 (1–4) ng/l, were similar across tertiles of hs-cTnI, including age, body surface area, LVEF, smoking status or an existing diagnosis of diabetes or hypertension (*P* > 0.05 for all). Patients in the highest tertile had a median (IQR) hs-cTnI concentration of 28 (24–35) ng/l before cycle 6 compared with 14 (12–20) and 6 (5–8) ng/l for the middle and lowest tertiles, respectively. There were no presentations of cardiac failure in this cohort during chemotherapy.

**Fig 3:**
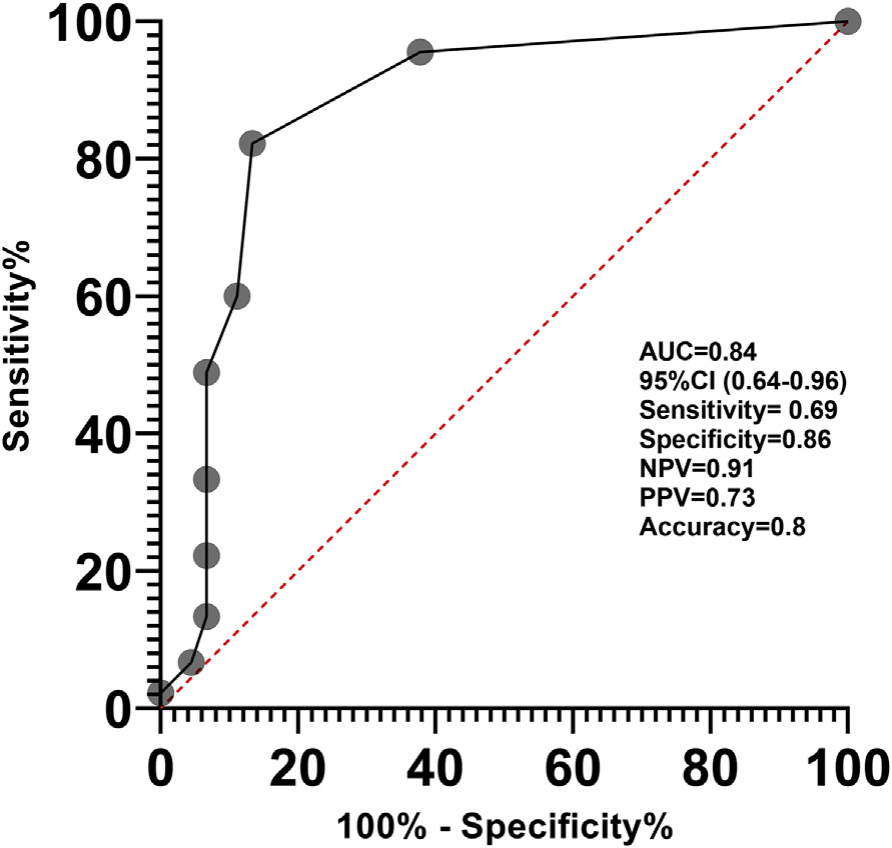
Receiver operator characteristics curve for the prediction of cardiac troponin I concentrations in T3 (upper tertile) before the sixth cycle, applying a threshold of 5 ng/l before cycle 2. AUC, area under the curve; CI, confidence interval; NPV, negative predictive value; PPV, positive predictive value.

Hs-cTnI concentration before the second treatment cycle was a strong predictor of subsequent myocardial injury (Figure 3). A threshold of ≥5 ng/l predicted subsequent development of troponin concentrations in the highest tertile before cycle 6, with a sensitivity of 69% and a specificity of 86% (c-statistic = 0.80; 95% confidence interval 0.64–0.96).

**Fig 4:**
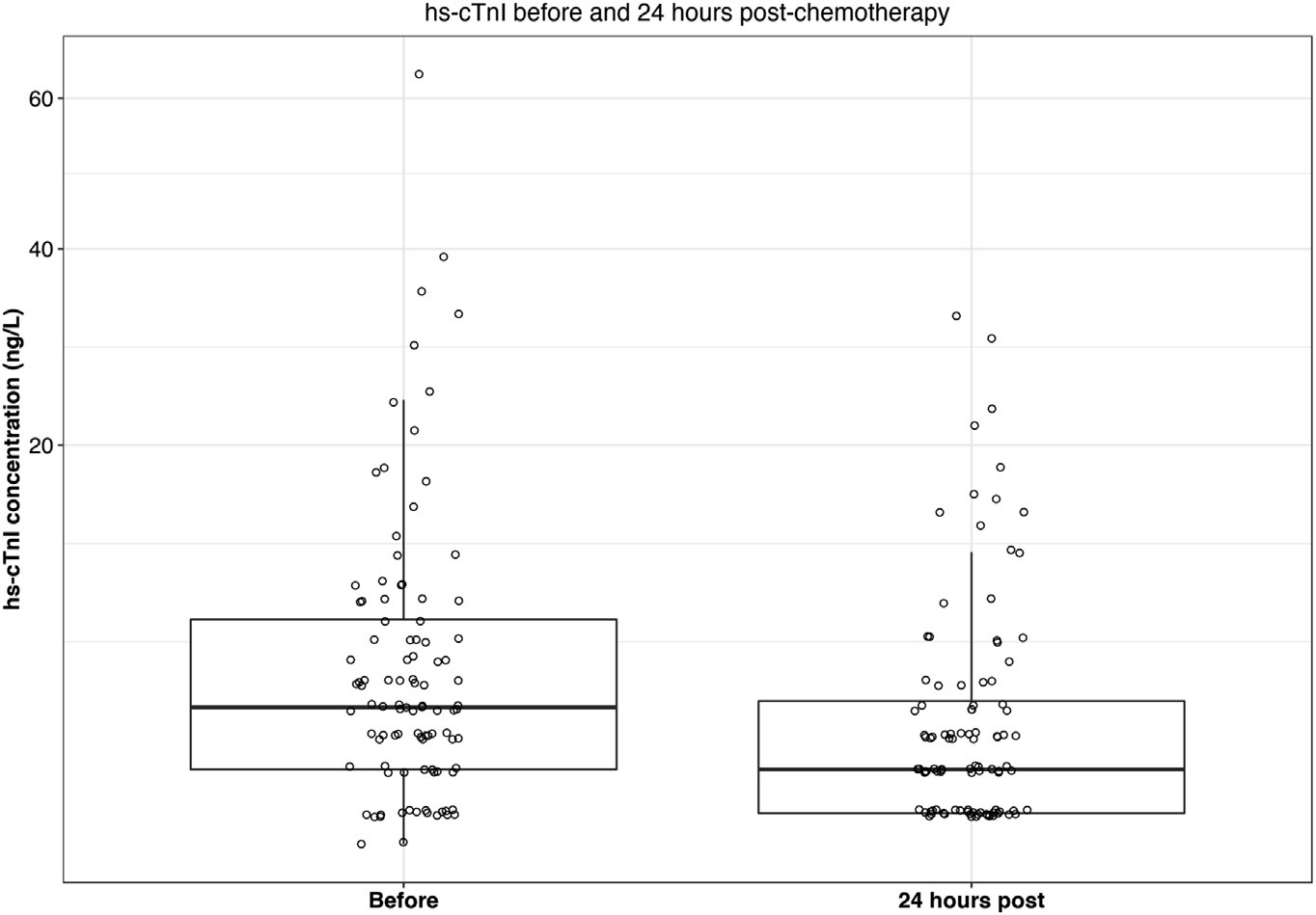
High-sensitivity cardiac troponin I (Hs-cTnI) concentration measured before and 24 h after each treatment cycle.

Compared with before chemotherapy, hs-cTnI concentrations fell by 33% (*P* < 0.001) 24 h after chemotherapy (Figure 4).

## Discussion

We have for the first time characterised the dynamic increase in hs-cTnI concentration occurring during anthracycline chemotherapy for breast cancer. Hs-cTnI assays are extremely sensitive, allowing for earlier and faster recognition of cardiotoxicity, offering clinicians a path to more rapidly implement cardioprotective treatment. The new assays are also precise, having small coefficient of variance levels below the 99% upper reference limit and a lower detection threshold when compared with contemporary assays [18].

Plasma hs-cTnI concentrations showed an anthracycline dose-dependent increase in accordance with the results of the PRADA study that used limited blood sampling before and after the completion of chemotherapy [17]. Using the same assay, the PRADA investigators showed linear correlation between baseline and post-chemotherapy hs-cTnI concentrations. For patients receiving 400 mg/m^2^ cumulative dose epirubicin, hs-cTnI rose from a median (IQR) of 1 ng/l (1–1.5) at baseline to 8 ng/l (4–14). Post-treatment hs-cTnI concentrations were significantly lower in patients receiving <400 mg/m^2^ cumulative dose. We quantified hs-cTnI concentrations before and 24 h after each anthracycline treatment to create a profile and identify the most suitable time of blood sampling during treatment. Unlike PRADA, our study was purely observational. By quantifying hs-cTnI concentrations before the next cycle of anthracycline chemotherapy we were able to identify an exponential increase for patients in the upper tertile with a median (IQR) hs-cTnI concentration of 28 (24–35) ng/l.

More than a third of patients developed biochemical evidence of sustained myocardial injury with plasma troponin concentrations above the 99th centile upper reference limit. Early changes in troponin accurately predicted final concentrations at the end of chemotherapy, suggesting that hs-cTnI represents a patient-specific marker anthracycline induced cardiotoxicity. By detecting myocardial injury after only a single treatment dose, troponin monitoring may allow early initiation of cardioprotective treatment in patients at greatest risk of cardiotoxicity.

Although it is notable that patients in the upper tertile exhibit a more obvious exponential troponin release, patients in the middle and lower tertiles also showed a degree of myocardial injury, albeit to a lesser extent. This first detailed profile of hs-cTnI concentrations supports the hypothesis that although there is substantial interindividual variation in sensitivity to the cardiotoxic effects of anthracycline therapy, this is not an idiosyncratic reaction.

Our observation that hs-cTnI concentrations fell 24 h after anthracycline administration was unexpected and suggests that myocardial injury is reliably determined from blood sampling carried out before rather than immediately after each treatment cycle. The mechanism explaining this early fall in hs-cTnI concentrations is uncertain but may be related to concurrent treatments including intravenous hydration and steroids administered with anthracycline during chemotherapy. Although this hypothesis remains to be tested, it illustrates the importance of understanding the kinetics of circulating troponin concentrations when selecting timepoints for monitoring cardiotoxicity.

In the absence of follow-up cardiac imaging, we were unable to assess the correlation between increasing troponin concentrations following anthracycline therapy and changes in cardiac function on serial imaging. Similarly, this study was underpowered to determine an association between troponin concentrations and clinical events, including death and heart failure. Nevertheless, the association between increased troponin concentrations and both left ventricular function and cardiovascular events has been well described in other contexts [[19], [20], [21]]. De Lemos *et al.* [19] categorised 3546 participants aged 30–65 years in a population-based cohort study according to hs-cTnT concentrations. The prevalence of left ventricular dysfunction (LVEF < 40%) increased from 0.005% in the lowest hs-cTnT category (<0.003 ng/ml) to 5.1% in the highest (≥0.014 ng/ml) (*P* < 0.001). During a median follow-up of 6.4 years, there were 151 total deaths, including 62 cardiovascular disease deaths. All-cause mortality increased from 1.9% to 28.4% across higher hs-cTnT categories (*P* < 0.001) [19]. After adjustment for traditional risk factors, hs-cTnT concentration remained independently associated with all-cause mortality (adjusted hazard ratio 2.8, 95% confidence interval 1.4–5.2 in the highest category). Cardiac troponin I concentrations predict the development of left ventricular dysfunction and congestive cardiac failure in patients receiving anthracycline containing high-dose chemotherapy. Using a contemporary assay with a detection threshold of 80 ng/l, Cardinale *et al.* [7] showed that patients with elevated concentrations during and after chemotherapy had much higher rates of heart failure and left ventricular dysfunction. After 3 years of follow-up, only 0.1% of patients with no cardiac troponin I concentration increase developed heart failure compared with 44% of patients exhibiting increased concentrations during and after chemotherapy [7].

Hs-cTnI may represent a better monitoring method compared with imaging measures of LVEF or myocardial strain, being relatively inexpensive, easier to perform, more sensitive and capable of detecting early subclinical cardiac injury.

In the oncology setting, previous studies with contemporary (non-high sensitivity) assays have shown the predictive value of elevated troponin concentrations for cardiotoxicity and its potential as a marker to identify high-risk patients warranting cardioprotective therapies [9,22]. Clinical trials investigating intervention and preventive strategies in anthracycline cardiotoxicity are challenging because the proportion of patients developing clinically relevant cardiac dysfunction is small. We believe subtle changes in troponin concentrations detected with high-sensitivity assay testing will identify high-risk patients early on during anthracycline treatment. This will identify a patient group in need of closer monitoring. This is particularly important if additional cardiotoxic treatment, such as trastuzumab, is planned. This approach is being adopted in the ongoing UK Cardiac CARE study randomising high-risk patients receiving anthracycline for breast and non-Hodgkin lymphoma to cardioprotective therapy.

Cardiac CARE participants will have hs-cTnI concentrations monitored before each cycle of chemotherapy. Patients with increased myocardial injury exhibiting upper tertile hs-cTnI concentrations will be randomly allocated to treatment with either a combination of angiotensin receptor blocker and B-blocker or standard care. The primary end point is change in LVEF on cardiac magnetic resonance imaging 6 months after the completion of chemotherapy.

## Conflicts of Interest

N.L. Mills has received honoraria from Abbott Diagnostics, Siemens Healthineers and Singulex; the University of Edinburgh has received research grants from Abbott Diagnostics and Siemens Healthineers. I.R. Macpherson has a consulting or advisory role with Daiichi Sankyo, Roche Products UK Ltd, Novartis Pharmaceuticals UK Ltd and Pfizer and also received travel, accommodation and expenses from Eisai and horonaria from Roche Products UK Ltd, Genomic Health and Novartis Pharmaceuticals UK Ltd. O. Oikonomidou has a consulting or advisory role for Pfizer, Roche, Astra Zeneca, EISAI and TESARO and has received travel/accommodation expenses and honoraria from Pfizer, EISAI, Roche, Elli-Lilly and Astra Zeneca.
